# Supplementary material for: Bandage: interactive visualization of de novo genome assemblies
Source: Bioinformatics. 2015 Jun 22;31(20):3350–2. doi: 10.1093/bioinformatics/btv383 (PMC4595904; doi:10.1093/bioinformatics/btv383)
Supplement: Supplementary Data [file supp_31_20_3350__index.html]

Bandage: interactive visualization of de novo genome assemblies — Bandage: interactive visualization of de novo genome assemblies — Supplementary Data 

# Bandage: interactive visualization of *de novo* genome assemblies

## Supplementary Data

files

- Supplementary Data - pdf file
