# Supplementary material for: Bandage: interactive visualization of de novo genome assemblies
Source: Bioinformatics. 2015 Jun 22;31(20):3350–2. doi: 10.1093/bioinformatics/btv383 (PMC4595904; doi:10.1093/bioinformatics/btv383)
Supplement: Supplementary Data [file supp_btv383_Bandage_supplementary_material.pdf]

BIOINFORMATICS APPLICATIONS NOTE

Bandage: interactive visualisation of *de novo* genome assemblies (supplementary material)

Ryan R. Wick [1], Mark B. Schultz [1], Justin Zobel [2] and Kathryn E. Holt [1]

[1]Department of Biochemistry and Molecular Biology, Bio21 Molecular Science and Biotechnology Institute, University of Melbourne, Parkville, Victoria, Australia
[2]Department of Computing and Information Systems, University of Melbourne, Parkville, Victoria, Australia

1 INTRODUCTION

De novo genome assemblers work by building an assembly graph. Different assemblers represent this graph in different ways, but they all contain sequences (which become assembled contigs) and connections between those sequences. In the past, assembly graphs have not been easily accessible, and most users only use the assembled contigs, not the graph. Bandage is a program for visualising assembly graphs. By displaying connections which are not present in the contigs file, Bandage opens up new possibilities for analysing *de novo* assemblies.

2 FEATURES

- Load multiple assembly graph formats: LastGraph (Velvet), FASTG (SPAdes) and Trinity.fasta.
- Position nodes automatically with an efficient graph layout algorithm.
- Zoom, pan and rotate the view using either mouse or keyboard controls.
- Reposition and reshape nodes by clicking and dragging with the mouse.
- Adjust graph scope: view the entire assembly graph or limit the visualisation to a region of interest.
- Copy node sequences to the clipboard or save them to file.
- Colour nodes using built-in colour schemes or user-defined colours.
- Label nodes using node number, length, coverage or user-defined labels.
- Find nodes quickly in a large graph using node numbers.
- Specify the thickness of nodes and allow thickness to reflect node coverage.
- Define the relationship between the length of a node and the length of its sequence.
- Draw graph in single node style: each node and its reverse complement appear as a single object.
- Draw graph in double node style: nodes and their reverse complements appear as separate objects with arrow heads to indicate direction.
- Highlight specific sequences with integrated BLAST search.
- Automatically determine contiguity to identify nodes that likely originated from the same segment of DNA.

3 USES

- Assess and compare the quality of assemblies quickly and visually.
- Identify problematic regions of an assembly.
- Resolve ambiguities in the graph to improve or complete *de novo* assemblies.
- Extract sequences for identification that are contiguous with a node of interest.
- Annotate graph images to illustrate assembly features.
- Manually reconstruct long sequences that extend through multiple nodes.
- Evaluate alternative splicing possibilities in assembled transcripts.

4 INSTALLATION

Bandage works equally well on Windows, OS X and Linux. Users are encouraged to download 64-bit binary executables using the links on http://rrwick.github.io/Bandage. No installation is necessary – just unzip and run. Windows and Mac binaries come packaged with all necessary libraries. The Linux binary has a couple of dependencies, specified in the zip file. Alternatively, you can clone the source code from GitHub and build Bandage yourself. The code and instructions are available here: https://github.com/rrwick/Bandage.

© Oxford University Press 2015.

5 EXAMPLE CASE

For a simple case, imagine a bacterial genome that contains a single repeated element in two separate places in the chromosome. A researcher (who does not yet know the structure of the genome) sequences it, and the resulting 100 bp reads are assembled with a *de novo* assembler.

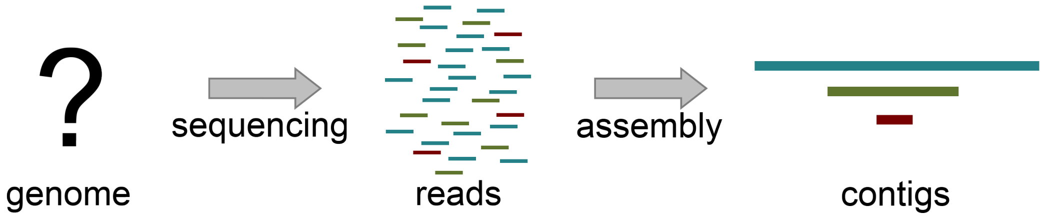

Because the repeated element is longer than the sequencing reads, the assembler was not able to reproduce the original genome as a single contig. Rather, three contigs are produced: one for the repeated sequence (even though it occurs twice) and one for each sequence between the repeated elements.

Given only the contigs, the relationship between these sequences is not clear. However, the assembly graph contains additional information which is made apparent in Bandage. There are two possible underlying sequences compatible with this graph: two separate circular sequences that share a region in common, or a single larger circular sequence with an element that occurs twice.

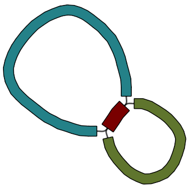

The assembly graph, as it appears in Bandage

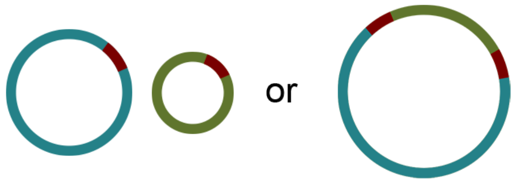

Possible underlying sequences compatible with the assembly graph

Additional knowledge, such as information on the approximate size of the bacterial chromosome, can help the researcher to rule out the first alternative. In this way, Bandage has assisted in turning a fragmented assembly of three contigs into a completed genome of one sequence.

6 SCREENSHOTS

These graphs are the same bacterial isolate assembled in SPAdes using three different k-mer values:

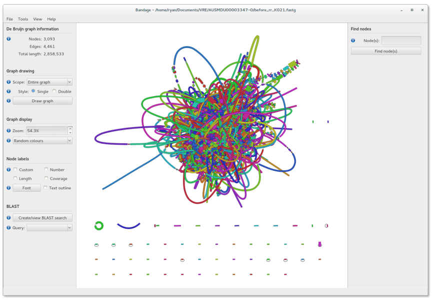

K-mer of 21. This value is too small, resulting in short contigs and many connections, giving a dense tangled graph.

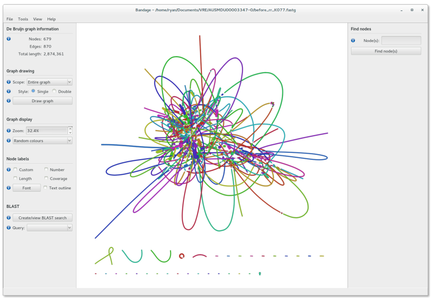

K-mer of 77. This is a good balance, giving a smaller number of long contigs that are well connected.

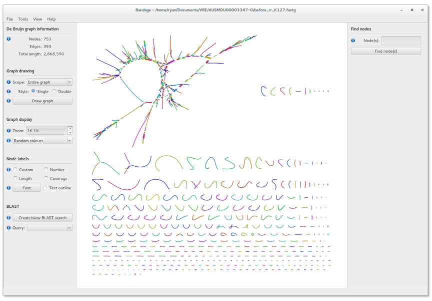

K-mer of 127. This value is too large, resulting in the graph breaking into many discontinuous pieces.

The following two images illustrate the difference between single and double nodes:

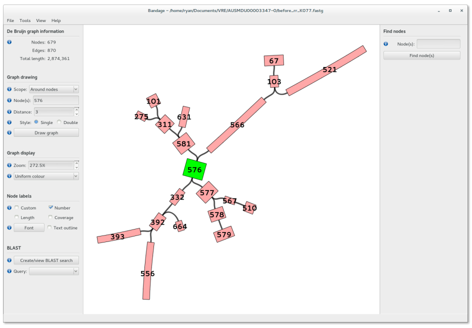

Single nodes, each holding both forward and reverse sequences.

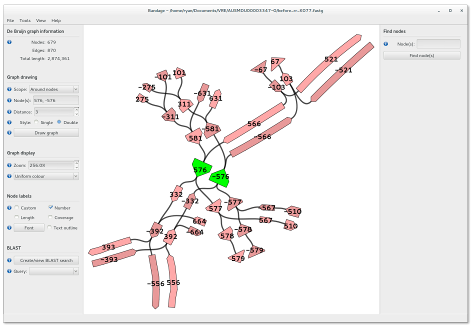

Double nodes, where forward and reverse nodes are drawn separately.

Examples of custom colouring and labelling of nodes in Bandage:

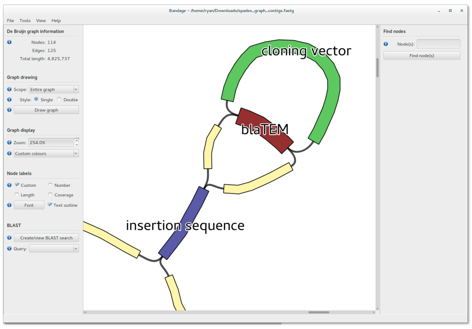

A bacterial transposon that shares a gene with the cloning vector.

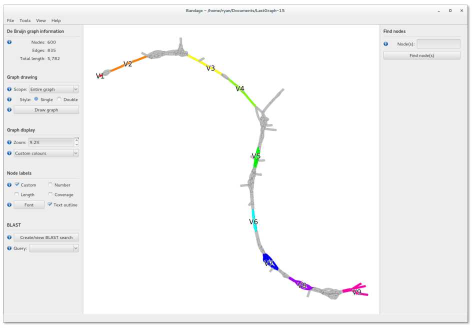

Assembly graph from metagenomic reads that align to particular 16S genes.

A very large graph from a metagenome assembly, displayed in Bandage:

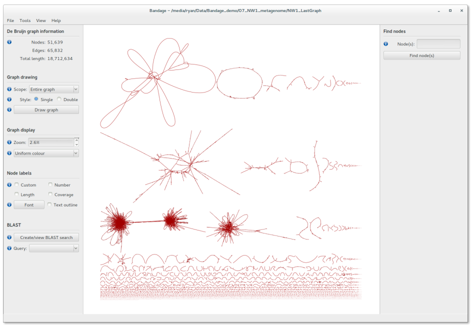

Zoomed out view, showing entire graph.

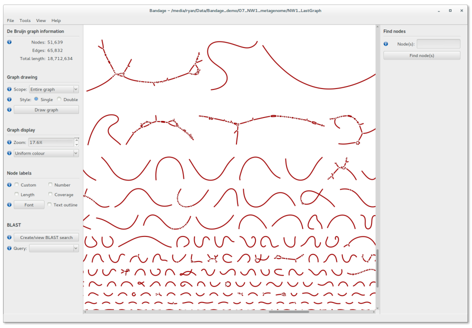

Zoomed in view of smaller subgraphs.

A BLAST search for a 58 kb transposon in a bacterial assembly:

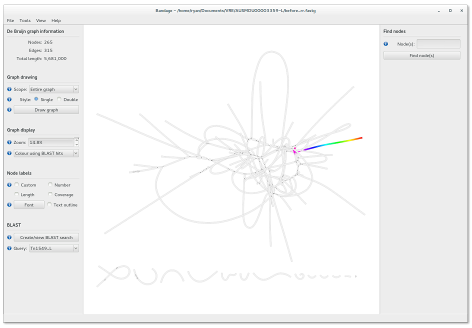

The entire assembly graph with the BLAST hits highlighted in colour.

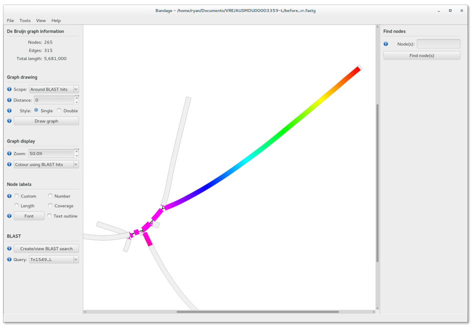

Reduced scope to only nodes containing BLAST hits.
